# Public understanding of climate change-related sea-level rise

**DOI:** 10.1371/journal.pone.0254348

**Published:** 2021-07-09

**Authors:** Rebecca K. Priestley, Zoë Heine, Taciano L. Milfont

**Affiliations:** 1 Centre for Science in Society, Victoria University of Wellington, Wellington, New Zealand; 2 School of Psychology, The University of Waikato, Tauranga, New Zealand; Technische Universiteit Delft, NETHERLANDS

## Abstract

Sea-level rise resulting from climate change is impacting coasts around the planet. There is strong scientific consensus about the amount of sea-level rise to 2050 (0.24–0.32 m) and a range of projections to 2100, which vary depending on the approach used and the mitigation measures taken to reduce carbon emissions. Despite this strong scientific consensus regarding the reality of climate change-related sea-level rise, and the associated need to engage publics in adaptation and mitigation efforts, there is a lack of empirical evidence regarding people’s understanding of the issue. Here we investigate public understanding of the amount, rate and causes of sea-level rise. Data from a representative sample of New Zealand adults showed a suprising tendency for the public to overestimate the scientifically plausible amount of sea-level rise by 2100 and to identify melting sea ice as its primary causal mechanism. These findings will be valuable for scientists communicating about sea-level rise, communicators seeking to engage publics on the issue of sea-level rise, and media reporting on sea-level rise.

## Introduction

Between 1902 and 2015, global sea level rose by 16 cm [1]. In recent decades sea-level rise has been accelerating, due to increasing rates of ice loss from the Greenland and Antarctic ice sheets. The majority of the world’s large cities are located on the coasts, and by 2050 one billion people could be at risk of coastal inundation or high-tide flooding [1]. While public understanding of climate change has been well canvassed in recent years [2–5], public understanding of sea-level rise is relatively unexplored.

Studies conducted in the United States and the United Kingdom show the public does not feel well informed about, and has difficulty understanding, sea-level rise associated with climate change [6, 7]. Moreover, a review of international sea-level rise communication [8] noted sea-level rise has suffered from low media attention and salience as a public issue. This lack of public understanding and salience as an issue is despite the fact that sea-level rise is already causing increased coastal flooding [9], and there is relatively low uncertainty about the amount of sea-level rise that will occur to 2050, which is projected to be between 0.24 and 0.32 m [10]. (Sea-level rise is always counted with respect to a reference date: in this paper we have adopted the baseline used in reports by the IPCC [11] and the New Zealand Ministry for the Environment [12]. Projections in these reports are relative to mean sea level averaged over the two-decade period 1986–2005.) Moreover, present day extreme water-level events will become commonplace within the next few decades [9] and sea-level rise of 0.39 m (0.26–0.53) by 2100 is likely even under the lowest emissions pathway (RCP2.6) [1]. While mitigation efforts are still important, adaptation to this coastal change is essential and urgent, and will require significant financial investments [9, 13]. Support from publics–as voters, taxpayers, coastal residents, and coastal business owners–is thus essential. This paper uses New Zealand as a case study to gauge the public’s current understandings of sea-level rise.

New Zealand is an island nation in the South Pacific, stretching 1,500 km between latitudes 34° and 47° south. The country has 18,000 km of coastline with no point on land more than 130 km from the sea [14], and it has a population of five million people [15], most of whom live in coastal cities or districts. Most New Zealanders believe climate change is occurring, is anthropogenic, and is a serious concern [3, 16]. Notably, stories about sea-level rise in the mainstream news media are largely consistent with the scientific consensus and reports issued by the Intergovernmental Panel on Climate Change (IPCC) [17].

In a 2015 report, the New Zealand Parliamentary Commissioner for the Environment noted, “It is certain that the sea is rising and will continue to do so for centuries to come. But much is uncertain–how rapidly it will rise, how different coastal areas will be affected, and how we should prepare” [18, p5]. The NZ SeaRise Programme aims to address this uncertainty with a 5-year (2018–2023) government-funded research initiative [19]. Its goal is to produce a set of location specific sea-level rise projections for New Zealand’s coastline, taking into account global and regional sea-level rise projections and new knowledge of local vertical land movements.

This study is part of the public engagement workstream of the NZ SeaRise Programme. Too often, development of outreach activities is dominated by intuitive approaches and personal or institutional motivations [20]. Relevant literature, though, advises that designing a public engagement campaign involves first understanding your audience and determining what they already know [21, 22]. Survey data is one form of evidence that can be used to inform the design of public enagement programmes on sea-level rise [23]. To gauge current understanding of sea-level rise, we therefore conducted a pilot study followed by a representative survey to identify what New Zealanders knew about the amount, rate and causal mechanisms of sea-level rise. The survey was initiated with an awareness that while media coverage in New Zealand tends to focus on IPCC projections and government reports, there is also voice given to climate change deniers and to people who warn of catastrophic sea-level rise [17]. Results of the survey will inform NZ SeaRise Programme researchers who are planning, or undertaking, efforts to engage publics with the issue of sea-level rise. Rather than starting with assumptions about what publics know and understand, researchers can use the survey results to approximate the knowledge and perspectives of the publics they are seeking to engage with.

Regarding the rate and timing of future sea-level rise, projections from the Fifth IPCC Assessment Report (AR5) put likely mean global sea-level rise by 2100 at between 0.26 to 0.98 m [11]. In 2017, the New Zealand Ministry for the Environment published projections for New Zealand of 0.46–1.05 m of sea-level rise by 2100, depending on how quickly global carbon emissions are reduced [12]. These projections did factor in a modest Antarctic ice melt contribution that was not assessed in the AR5; however, a subsequent study using a structured expert judgment approach that evaluated all the latest projections [24] found that global sea-level rise could exceed 2 m by 2100 under a low-probability, high-emissions scenario. The 2019 IPCC Special Report on the Oceans and Cryosphere in a Changing Climate [1], which reviewed more recent studies [25–27] than did AR5, assessed likely global sea-level rise by 2100 as ranging from 0.29 m to 1.10 m under the different IPCC scenarios. In the same report, thermal expansion, melting of the Greenland ice sheet, and melting of land-based glaciers were identified as the main contributors to sea-level rise to date, with greater contributions from melting of the Antarctic ice sheets for the rest of the century and beyond [10].

To understand the public’s knowledge about the amount, rate and causal mechanisms of sea-level rise, we commissioned a national survey. To reflect the New Zealand population, survey responses were weighted by age, sex, highest education, ethnicity and region using census data. The percentage results shown below are thus weighted to be representive of the New Zealand population.

## Method

Data were collected from a representative sample of 1100 New Zealanders aged 18 and over conducted by Horizon Research in September 2019. Participants, who were part of the company’s research panel, completed 15 multichoice questions plus demographic questions online in their own time. In the information provided at the start of the survey, participants were advised that by continuing the survey they were giving consent for their responses to be used in this research, and were assured that it was an anonymous survey and they would not be identifiable. The research was approved by the Human Ethics Committee at Victoria University of Wellington in August 2019 (reference number 0000027157).

The majority of respondents were male (50.5%), within the 45–54 years age bracket (22.7%), with a household income between $70,001 and $100,000 per year (17.9%), employed (69.5%), and indicated a personal income between $30,001 and $50,000 per year (19.6%), an undergraduate (Bachelor) degree as the highest qualification (29.1%), Auckland Council as their local government (32.8%), and NZ European/Pakeha as their self-identified ethnicity (64.2%).

Prior to the representative survey, a pilot online survey, comprising 27 multi-choice and short answer questions plus demographic questions, was conducted in July 2019 and received 665 respondents, with respondents aged between 18 and 87, most of whom (58%) were female.

The pilot survey was useful for informing the final representative survey and helping us to select the 15 most critical questions (scaling down the initial survey was necessary because of the financial constraints involved in using a company to collect data). We acknowledge Akerlof’s points that “some fraction of the public has no prior opinion on questions asked in surveys, but feels impelled to provide an answer” [23, p. 423] and that “reported beliefs and attitudes may either be randomly chosen by the respondent or selected based on cues from the question format” [23, p. 423]. We excluded ‘I don’t know’ answers in many of the response options, though, because we wanted participants to indicate their view, or best approximate, even if they didn’t have full knowledge of the topic, and also because ‘I don’t know’ response options can yield unwanted methodological issues [28].

The S1 File provides more detail about survey design and findings from other questions included in the survey (S1 File–representative and pilot surveys). The full survey, raw data, as well as overall descriptive results produced by Horizon Research, can be obtained in the Open Science Framework: https://osf.io/vcjuf/.

### Public overestimates sea-level rise possible by 2100

We first examined New Zealanders’ understanding of current sea-level rise. As noted, average global sea level has risen by more than 16 cm since 1900 [1]. Respondents were thus asked to identify, from options ranging from ‘not at all’ to ‘more than 40 cm’, how much they thought global sea level has risen since 1900. More than a quarter of respondents answered ‘10–20 cm’ (26.9%), a figure that is in line with current understanding of sea-level rise to date. But a larger proportion (38.2%) of respondents either *underestimated* the amount of sea-level rise, selecting ‘not at all’ (11.2%) or ‘5–10 cm’ (27%), or *overestimated* (35%) the amount of sea-level rise from 1900 to date, selecting ‘20–30 cm’ (13.4%), ‘30–40 cm’ (8.4%) or ‘more than 40 cm’ (13.2%).

**Fig 1:**
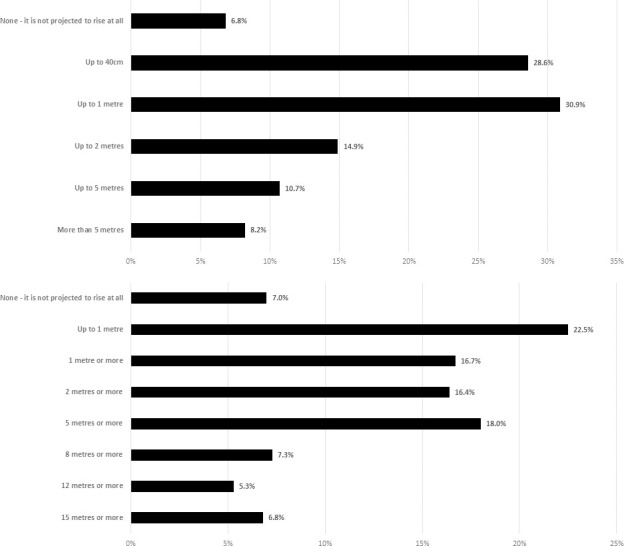
Sea-level rise projections to 2100. (a): Respondents were asked ‘How much do you think global sea level is projected to rise by 2100?’ (b): Respondents were asked ‘How much could global average sea level rise under ‘a scientifically credible worst-case scenario’?.

We then examined knowledge of sea-level rise projections to 2100 (see Fig 1A). While a minority (6.8%) of respondents said that sea level was not projected to rise by 2100, nearly 75% of respondents selected options that were in line with scientifically-plausible projections, selecting ‘up to 40 cm’ (28.6%), ‘up to 1 m’ (30.9%), and ‘up to 2 m’ (14.9%). But a large group of respondents (18.9%) *overestimated* sea-level rise projections to 2100, selecting ‘up to 5 m’ (10.7%) or ‘more than 5 m’ (8.2%).

To further explore the amount of sea-level rise respondents thought was possible by 2100, we asked how much global sea level could rise by 2100 under ‘a scientifically credible worst-case scenario’ (see Fig 1B). While a similar small number of respondents said that sea-level rise was not going to happen at all (7%), and 22.5% of respondents *underestimated* the worst-case scenario by selecting ‘up to 1 metre’, this time only 33.1% of respondents gave an answer broadly in line with current science, answering ‘1 m or more’ (16.7%) or ‘2 m or more’ (16.4%). Another 37.4% of respondents substantially *overestimated* sea-level rise by 2100, selecting ‘5 m or more’ (18%), ‘8 m or more’ (7.3%), ‘12 m or more’ (5.3%) or ‘15 m or more’ (6.8%).

To assess what respondents thought was physically possible, we then asked what they thought would be the ‘maximum amount of sea-level rise’ that could occur if ‘all of the glaciers and ice sheets and ice caps on planet Earth melted’, and what was the fastest time period over which this could occur. The largest group (38.8%) of respondents *underestimated* the maximum amount of sea-level rise possible, selecting ‘about 30 m’. The next largest group (22.3%) chose a figure broadly in line with current estimates of 66.07 m [29], selecting ‘about 60 m’. The remaining 38.9% of respondents *overestimated* total maximum sea-level rise, selecting ‘about 120 m’ (17%), ‘about 240 m’ (10.9%) and ‘more than 500 m’ (11%).

In selecting the ‘fastest period of time’ over which their perceived maximum amount of sea-level rise could occur, respondents *overestimated* the speed at which all the planet’s ice could melt. While scientists suggest that such melting could take place under a sustained warming climate over a period of thousands of years [30, 31], the majority of respondents overestimated how fast the planet’s ice could melt, selecting ‘decades’ (33.1%) or ‘centuries’ (39%). A smaller group (11.6%) of respondents answered in line with the science by selecting ‘thousands of years’. The remaining respondents selected ‘tens of thousands’ (3.2%) or ‘hundreds of thousands’ (2.7%) of years, or acknowledged that ‘I really don’t know’ (10.4%).

### Public identified melting sea ice as causing sea-level rise

**Fig 2:**
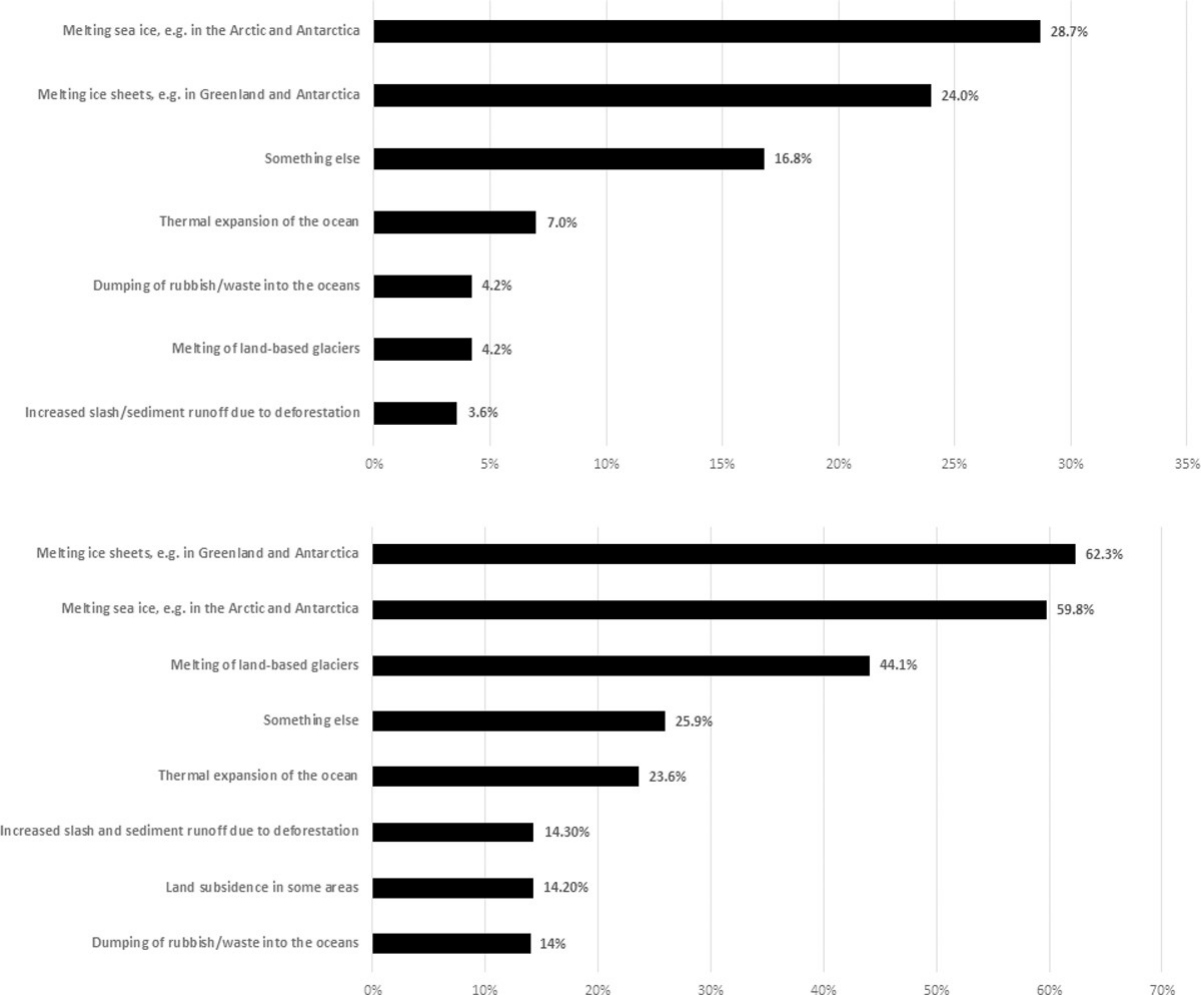
Major contributors to sea-level rise. Respondents were asked to identify and rank the major contributors to sea-level rise. In (a) we show respondents’ top ranked cause of sea-level rise (causes selected by fewer than 2% of respondents are not shown). In (b) we show the sum of the top three causes identified by respondents (causes ranked by fewer than 10% of respondents are not shown).

Respondents were also asked to identify and rank the major causes of sea-level rise from a ten-item list that included melting ice sheets, melting land-based glaciers, melting sea ice, thermal expansion, and land subsidence. No definitions of these phrases were provided as our intention was to gauge people’s understanding of sea-level rise from existing sources of information, such as the media. While climate scientists and the media make efforts when communicating with publics to *not* identify melting of Arctic and Antarctic sea ice (which is widely covered in the media) as causing sea-level rise, 28.7% of respondents identified this as their top ranked cause of sea-level rise (see Fig 2A). Encouragingly, other respondents’ top ranked causal mechanisms that *do* contribute to sea-level rise, selecting melting ice sheets (24%), thermal expansion of the oceans (7%), and melting of land-based glaciers (4.2%).

When we grouped respondents’ top three rankings for causes of sea-level rise (Fig 2B), melting ice sheets (62.3%) was first, followed by melting sea ice (59.8%), melting of land-based glaciers (44.1%), thermal expansion of the oceans (23.6%) and land subsidence in some areas (14.2%). There were also large proportions of respondents ranking some other mechanisms that do not impact on sea-level rise, such as ‘increased slash and sediment runoff due to deforestation’ (14.3% ranked it in their top three), ‘dumping of rubbish/waste into the ocean’ (14%), ‘increased rainfall in the tropics’ (9.9%) and ‘submarine landslides’ (7.5%).

Notably, 25.9% of respondents also ranked ‘something else’ as one of the top three causes of sea-level rise. When asked to specify what these causes might be, more than half (54%) were unsure or gave no answer, while 23.5% attributed sea-level rise broadly to climate change, global warming or human activities, and 16.5% gave answers such as ‘solar activity’, ‘earthquakes’, or ‘storms’, suggesting these respondents believe natural processes are responsible for sea-level rise.

### Challenges of projecting and communicating sea-level rise

Projecting sea-level rise is complicated [32]. Different climate, mountain glacier and polar ice sheet models produce different results. This is especially the case for Antarctic ice sheet models, which can range from projecting a net gain in mass to a significant loss. Thus, the IPCC approach is to take the mean of ensembles of model experiments, that all use broadly similar boundary conditions and climate forcings. Moreover, glacier melting, vertical land movement, gravitational effects, and ocean dynamics all contribute to sea-level rise being different in different locations; and calculations must be made for a number of different scenarios representing different mitigation pathways [33]. Sea-level rise projections are therefore usually scenario dependent and framed in terms of a probablistic range, rather than a single figure, to reflect the uncertainty in the projections.

Communicating about sea-level rise, given the uncertainties inherent in projections, is also complicated [8]. IPCC reports, which assess and interpret all the latest science, contain jargon and caveats about ‘uncertainty ranges’ with evidence that publics misunderstand verbal probability expressions, known as calibrated uncertainty language [34], such as ‘likely’ and ‘unlikely’ [35]. Even the IPCC summaries for policymakers have been criticised for their formal structure and use of jargon [36]. This is a complex challenge for science communicators. As noted by Seethhaler et al, communicating about uncertainty is “one of the greatest challenges in science communication, one that raises ethical issues including, but not constrained to, finding a middle ground in prognostication between false assurances and doomsday scenarios” [37, p. 381].

Media interpretations of the science adds to the problem. While the New Zealand media gives extensive coverage to IPCC projections, it also gives voice to more extreme and catastrophic views from local and international scholars. For example, in 2016 New Zealand media reported on research that projected several metres of sea-level rise by 2100 [38–40], and in 2017 there was significant media coverage when a philosophy professor warned that sea-level rise could reach 5 m or more by 2100 [41–43]. These stories, though, also included comments from scientists who communicated the IPCC projections and from politicians who underestimated the importance of sea-level rise as an issue. Such indiscriminate media coverage, and attempts at journalistic balance, draw the uncertain nature of sea-level rise projections to the public’s attention and detract from the certainties that exist out to 2050. This could therefore be contributing to public confusion on the issue. While our pilot study revealed that the majority of respondents (68%; n = 518) felt somewhat or pretty well informed about sea-level rise, only 38% (n = 519) felt somewhat or extremely clear about the rate/timing/extent of sea-level rise to 2100.

Some of the most extreme projections selected by our respondents go beyond the 5 m of sea-level rise by 2100 that some media have reported. For example, the 6.8% of respondents who thought sea-level rise could reach ‘15 m or more’ by 2100 and the 33.1% who believed that all the planet’s ice could melt over a period of ‘decades,’ selected options that are unprecedented in the geological record and defy physical laws around how fast ice can melt, even under extreme temperature forcing [44, 45].

Beyond the more descriptive analysis of the answers, we also examined the extent to which overestimation was associated with level of concern about sea-level rise. Results from *t*-tests confirmed that respondents who were more likely to overestimate the amount of sea-level rise and how fast this would occur were significantly more likely to express greater concern about sea-level rise to 2100 in New Zealand, and globally, compared to their counterparts (i.e., *t* > 5, *p* < .001; Cohen’s *d* > .50; see Section 1.4 of the S1 File for detail).

When assessing the severity of environmental problems for geographically distant places, individuals tend to rate the severity of these problems as worse ‘there’ than ‘here’ [46–48]. Hence, we also examined whether such spatial bias would be observed in relation to concern for sea-level rise. The spatial bias effect was operationalized as the difference between ratings of national concern and global concern (i.e., average Aotearoa New Zealand minus average global). A difference score equal to zero represents judgement of no nation–global distinction, while values other than zero represent biased comparative judgements. Values above zero represent the expected spatial bias effect with global sea-level rise judged as more concerning than national sea-level rise, and values below zero represent an opposite effect. For most respondents (78.8%) a biased judgement was absent (i.e., the difference score was zero) but results from a one-sample *t*-test indicate the mean difference score of .08 (*SD* = .59) was statistically different from zero, *t*(1034) = 4.28, *p* < .001, Cohen’s *d* = .14. A spatial bias effect was thus observed for sea-level rise: respondents judge global sea-level rise as more concerning than national sea-level rise.

The public’s association of melting sea ice with sea-level rise, as well as its selection as the top-ranked cause of sea-level rise, provides further evidence of public confusion on this issue, which may be due to the significant media coverage given to melting sea ice in the Arctic. It is possible, though, that some respondents might have been influenced by science that says the flow of ice sheets can be modulated by the presence or absence of sea ice [49–51].

Research into how publics understand sea-level rise is still an emerging field [8] and there are few international studies with which to compare our results. However, we note that our findings that respondents tend to *overestimate* the amount of sea-level rise possible, contrast with a 2019 Australian study showing that New South Wales coastal users and businesses tended to *underestimate* the amount and rate of sea-level rise along the NSW coast with 45% of respondents predicting a rise of 1–25 cms in the next 20–50 years [52]. Similarly to our results a 2016 American study found that individuals were concerned by the impacts of sea-level rise but were uncertain about its timing [53]. This survey did not ask respondents to estimate the projected amount of sea-level rise, but asked “When do you believe the effects of sea-level rise will significantly impact the county, if ever?” Finally, a 2016 study of residents of New Jersey following Hurricane Sandy showed that while participants believed sea-level rise was occurring, they did not view sea-level rise as a current threat to themselves [54]. This survey did not ask about the projected amount of sea-level rise or its timing, but asked participants whether they believed sea-level rise was occurring and whether it was affecting them. These studies and our own show that there is plenty of space to develop more in-depth understanding of how the public perceives the threat of sea-level rise.

The present research revealed the unexpected finding that one key audience for public engagement with sea-level rise is the concerned public who *overestimate* sea-level rise by 2100 as being catastrophic (and scientifically implausible). While there are obvious dangers associated with a public that underestimates or minimises the dangers of sea-level rise–such as not taking adaptation measures, or not supporting governments committed to mitigation and adaptation–there are also dangers associated with a public that *overestimates* sea-level rise. A focus on extreme (and often unsound) projections of sea-level rise can result in public anxiety and feelings of helplessness, rather than motivation to take action to mitigate and adapt [55, 56]. Nevertheless, the present research shows that publics are aware of, and concerned about, 21^st^ century sea-level rise, which is already impacting on coastal dwellers and infrastructure. Any public engagement efforts will therefore need to be more focused and nuanced than raising awareness of the issue, and could include, for example, provision of ongoing and credible sea-level rise information that is accessible to a wide range of publics and decision-makers. With the knowledge that overestimating can lead to failure to act, communicating with this segment of the audience could focus on providing scientifically plausible projections of sea-level rise and communicating that both adaptation and mitigation–to avoid the most extreme sea-level rise scenarios–are necessary.

## Conclusion

As noted by Akerlof [23] in relation to a project focused on community adaptation to sea-level rise, ‘communication programs and public consultation by governments can benefit from the use of survey data to support evidence-based decision-making’ (p 406). Overall, our survey findings indicate that New Zealanders have a tendency to *overestimate* the amount of sea-level rise possible by 2100, with those more likely to overestimate future projections tending to be most concerned about sea-level rise. They also have a mistaken association of sea-level rise with melting of sea ice, and judge global sea-level rise projections as higher, and more concerning, than national sea-level rise projections.

Criticisms of the ‘deficit model’ of science communication show that encouraging action on an issue–such as sea-level rise–is not as simple as ensuring that publics are fully informed [57]. However, it is essential that publics have access to information that can inform their decision making, particularly with an issue such as sea-level rise that has widespread consequences for coastal livelihoods. If publics have a better understanding of the scale and rate of sea-level rise projected over this century and beyond, and the adaptation measures necessary to respond to this, there is evidence they will be more willing to also adopt mitigation measures [58]. Hence, having publics informed about sea-level rise and its impact will enable them to be involved in decisions about both adaptation and mitigation strategies; that is, to prepare for the 0.24 to 0.32 m of sea-level rise we know is coming by 2050, as well as to reduce greenhouse gas emissions to avoid the higher sea levels possible by 2100 and beyond. The inclusion of informed publics in these decisions (and discussions) is an essential part of democratic decision making [59].

## Supporting information

S1 File Representative and pilot surveys. Here we provide more detailed information about survey design and results.
(DOCX)
