# Supplementary material for: Public understanding of climate change-related sea-level rise
Source: PLoS One. 2021 Jul 9;16(7):e0254348. doi: 10.1371/journal.pone.0254348 (PMC8270426; doi:10.1371/journal.pone.0254348)
Supplement: S1 File — Here we provide more detailed information about survey design and results. (DOCX) [file pone.0254348.s001.docx]

**S1 File: Representative and pilot surveys**

**1.0 Representative survey**

The full survey, as well as overall descriptive results produced by Horizon Research, and the raw data, can be found in the Open Science Framework: https://osf.io/vcjuf/.

**1.1 Rationale for Main Questions**

The survey included 15 multichoice or ranking questions and additional demographic questions; we discuss six of the multichoice or ranking questions in the article. Table S1 lists these multichoice questions and references the correct ranges used in our interpretation of the findings.

**Table S1. Scientific references for the sea-level rise quantification questions discussed in the article.**

| Survey question | Correct figures |
| --- | --- |
| *How much has sea level risen since 1900?* | 0.16 m (0.12–0.21) 1902 – 2015. [1, p.10]  0.16 m (0.12 to 0.21) 1902 – 2010. [2, p. 334]  An often-cited figure is 0.21 m between 1880 – 2009. [3, p. 585] |
| *How much global sea level is projected to rise, on average, by 2100?* | 0.43 m (0.29 to 0.59) RCP2.6. [2, p. 352]  0.55 m (0.39 to 0.72) RCP4.5. [2, p. 352]  0.26 m (.12 to .53). [4, p. 11197]  0.44 m (0.28 to 0.61) RCP2.6. [5, p. 1182]  0.53 m (0.36 to 0.71) RCP4.5. [5, p. 1182]  0.55 m (0.38 to 0.73) RCP6.0. [5, p. 1182]  0.78 m (Low 1) and 0.83 m (Low 2). [6, p. 1342] |
| *Under a worst-case scenario, how much global sea level could rise, on average, by 2100? (m)* | 0.74 m (0.52 to 0.98) RCP8.5. [5, p. 1182]  0.84 m (0.61 to 1.10) RCP8.5. [2, p. 352]  0.5 to 1.2 m RCP8.5. [7, p. 383]  5 metres by 2100 with scenarios of 5 year and 10 year doubling of ice melt. [8, p. 3767]  0.51 m (0.22 to 1.13) H scenario roughly equal to RCP8.5. [4, p. 11197]  2 m + within the 90% uncertainty bounds of H scenario [4, p. 11195]  2 m. [6, p. 1342] |
| *If all of the glaciers and ice sheets and ice caps on planet Earth melted, what is the maximum amount of sea level rise that this could cause?* | ~ 66.07 m (Contributions approximate as Antarctic ice sheet 58.3 m, Greenland 7.36 m, glaciers 0.41 m). [9, , p. 321] |
| *(in reference to above) how fast could this happen* | Antarctic contribution of 9.31 m by 5000 CE under RCP 8.5. [10, p. 421]  Antarctic contribution of > 15 m of GMSL rise in the next 500 years. [11, p. 596]  Kinematic max of 2 m per century suggests it would take >3000 years to melt all the ice on earth. [6] |

**1.2 Aotearoa New Zealand vs global sea level rise**

The results reported in the main paper are in response to questions about global sea-level rise. As well as asking about the *global* context (see Table S1) we asked about sea-level rise in Aotearoa New Zealand by asking the same questions but substituting ‘global’ with ‘Aotearoa New Zealand’.

Respondents thought that sea-level rise since 1900 was less in Aotearoa New Zealand than globally. 35% of respondents selected 20 cm or more for global sea level rise since 1900, while 28.1% selected 20 cm or more for Aotearoa New Zealand,

When asked about how much global sea-level rise is projected by 2100, 18.9% of respondents selected options of ‘up to’ or ‘more than’ 5 m compared to only 15.1% of respondents who selected these options for Aotearoa New Zealand.

Respondents also thought that under a scientifically credible worst-case scenario, sea-level rise in Aotearoa New Zealand would be less than global sea-level rise. For the global question, 37.4% selected options of 5 m or higher, whereas only 30.5% of respondents selected these options for New Zealand.

While respondents were more likely to be ‘very concerned’ about global sea-level rise (29.7%) than about sea-level rise projected for Aotearoa New Zealand (24%), this was balanced by the responses for ‘concerned’ of 31.7% and 37.3%, respectively.

Respondents were also asked if they had been affected by sea-level rise in any way. Here, only 6% of respondents said ‘yes’. But when asked ‘Have you taken any actions in your personal or professional life to mitigate or adapt to sea-level rise in any way?’, 13.2% of respondents said ‘yes’.

**1.3 Belief questions about causes of climate change and sea-level rise**

Beyond the questions noted above, the survey included belief questions about climate change and sea-level rise. To measure and analyse opinions about the causes of climate change, we followed the approach taken by Hornsey, Harris, and Fielding (2018) [12].^[[1]](#footnote-1)^ We treated answers to this question as a continuous measure of climate change scepticism by coding ‘entirely caused by human activity’ as 1, ‘mainly caused by human activity’ as 2, ‘partly caused by natural processes and caused partly by human activity’ as 3, ‘mainly caused by natural processes’ as 4, and ‘entirely caused by natural processes’ and ‘I think there is no such thing as climate change’ as 5. Participants who selected ‘Don’t know’ or ‘No opinion’ were excluded from the analyses.

We also used a similar single-item measure for opinion about causes of sea-level rise. We coded ‘sea-level rise is entirely caused by human induced climate change’ as 1, ‘sea-level rise is mainly caused by human induced climate change’ as 2, ‘sea-level rise is partly caused by natural variation and partly by human induced climate change’ as 3, ‘sea level rise is mainly cause by natural variation’ as 4, both ‘sea-level rise is entirely caused by natural variation’ and ‘sea-level is not rising’ as 5, and ‘Don’t know’ and ‘No opinion’ as missing.

In response to these questions, New Zealanders seem to fit into three groups: convinced, neutral, or sceptical. The convinced respondents identified climate change as being ‘mainly’ (31.3%) or ‘entirely’ (8.6%) due to human activities and sea-level rise being ‘mainly’ (27.9%) or ‘entirely’ (9.8%) a result of human-induced climate change. Neutral respondents believed climate change was ‘partly caused by natural processes and partly by human activity’ (33%), or were ‘not sure’ (4.1%) or did not have an opinion (2.7%) on the causes of climate change. The neutral respondents similarly thought sea-level rise was ‘partly caused by natural variation and partly by human induced climate change (34.2%) or were ‘not sure’ (5.3%) or did not have an opinion (3.2%) on the causes of sea-level rise. A very small group seemed to be climate change deniers, saying that climate change is entirely caused by natural processes (5.2%), or there is ‘no such thing’ as climate change (1.7%) and that sea level is not rising (5.2%) or is entirely caused by natural variation (5.3%).

As noted in section 1.2, respondents were ‘very concerned’ (29.7%) or ‘concerned’ (31.7%) about sea-level rise projected to happen globally to 2100. A further 18.6% of respondents felt ‘neutral’ about it, or were ‘not very concerned’ (8%), ‘not concerned at all’ (6.8%) or were ‘not really sure’ (5.2%).

As expected, the questions about causes of climate change and sea-level rise were highly correlated (*r* = .758, *p* < .001; *N* = 980). Notably, belief in anthropogenic sea-level rise was highly correlated with concern about sea-level rise projected to happen over the period to 2100 in Aotearoa New Zealand (*r* = .509, *p* < .001; *N* = 988) and globally (*r* = .532, *p* < .001; *N* = 1035); and these concern questions were highly correlated (*r* = .875, *p* < .001; *N* = 1035).

**1.4 Results from independent samples *t*-tests**

As described in the main article, when asked ‘Under a scientifically credible worst-case scenario, how much do you think global sea levels could rise by 2100?’ 6.8% of respondents thought sea-level rise could reach ‘15 m or more’ by 2100. When asked ‘What do you think is the fastest period of time over which [the maximum amount of sea-level rise] could occur?’ 33.1% believed that all the planet’s ice could melt over a period of ‘decades’.

We dummy coded these two questions to compare participants who selected ‘15 m or more’ or ‘decades’ (coded as 1) to those who selected other answers to the questions (coded as 0). We then conducted *t*-tests to examine whether the participants who selected these extreme answers were more concerned about sea-level rise to 2100 compared to participants who selected distinct answers to the two questions. For these analyses, the ‘I’m not really sure’ answers to the concern questions were treated as missing.

We only report the summary statistics in the main article (i.e., *t* > 5, *p* < .001; Cohen’s *d* > .50), but Tables S2 and S3 below provide the full results of these analyses. As can be seen, respondents who were more likely to overestimate the amount of sea-level rise and how fast this would occur were significantly more likely to be express greater concern about sea-level rise to 2100 in New Zealand and globally compared to their counterparts.

**Table S2. *T*-test for the question ‘how much do you think global sea levels could rise by 2100?’**

|  | Selected ‘15 m or more’  (*n*=74) | Selected another answer  (*n*=961) |  |  |  |  |  |  |
| --- | --- | --- | --- | --- | --- | --- | --- | --- |
|  | *M* (*SD*) | *M* (*SD*) | Mean Diff. | 95% CI  [Lower, Upper] | *t* | df | *p* | Cohen’s *d* |
| How concerned do you feel about the sea level rise projected to happen in Aotearoa New Zealand over the period to 2100? | 1.66  (.91) | 2.40 (1.17) | .75 | [.47, 1.02] | 6.64 | 93.04 | <.001 | .71 |
| How concerned do you feel about the sea level rise projected to happen globally over the period to 2100? | 1.68  (.94) | 2.32 (1.20) | .64 | [.41, .87] | 5.50 | 92.78 | <.001 | .59 |

Note. Self-rated concern levels range from 1 (Very concerned) to 5 (Not concerned at all). The ‘I’m not really sure’ answers were treated as missing.

**Table S3. *T*-test for the question ‘what is the fastest period of time over which [the maximum amount of sea level rise] could occur?’**

|  | Selected ‘decades’ (*n*=348) | Selected another answer  (*n*=688) |  |  |  |  |  |  |
| --- | --- | --- | --- | --- | --- | --- | --- | --- |
|  | *M* (*SD*) | *M* (*SD*) | Mean Diff. | 95% CI  [Lower, Upper] | *t* | df | *p* | Cohen’s *d* |
| How concerned do you feel about the sea level rise projected to happen in Aotearoa New Zealand over the period to 2100? | 1.91  (.96) | 2.57 (1.20) | .66 | [.53, .80] | 9.66 | 844.97 | <.001 | .61 |
| How concerned do you feel about the sea level rise projected to happen globally over the period to 2100? | 1.80 (1.01) | 2.51 (1.21) | .71 | [.57, .85] | 9.96 | 815.67 | <.001 | .64 |

Note. Self-rated concern levels range from 1 (Very concerned) to 5 (Not concerned at all). The ‘I’m not really sure’ answers were treated as missing.

**2.0 Pilot Study**

The survey described in the main article was informed by a pilot study, comprising 27 multi-choice and short answer questions plus demographic questions, completed online in July 2019 by 665 respondents, aged between 18 and 87, most of whom (58%) were female. This survey was distributed online and by email, through our own networks, and had an unrepresentative skew towards well educated (56.1% had some form of postgraduate qualification), high income earners (22.39% earned NZD100,000 or more), living in the capital city of Wellington (34.12%).

A full copy of the pilot survey is available in the OSF page of the project: https://osf.io/vcjuf/. Here we present a summary of the key results of the pilot survey.

**2.1 Information about sea-level rise**

We asked respondents where they got information about sea-level rise. The top ten most selected options, from a list of 14 provided, were: (1) International news media - online and print editions; (2) New Zealand newspapers - online and print editions; (3) Stories shared on social media; (4) Radio New Zealand; (5) Public lectures and talks; (6) Directly from scientists; (7) Television; (8) From local councils or government agencies; (9) New Zealand Magazines - online and print editions; and (10) Classes or lectures at school or university.

Respondents were then asked how well informed they felt about sea level rise, how informed they felt about how it might impact on Aotearoa New Zealand, and how clear they felt about the amount and rate and timing of sea-level rise that will impact Aotearoa New Zealand to 2100. Responses are shown in the figures below.

**Figure S1: ‘How informed do you feel about sea level rise?’ (n=518)**


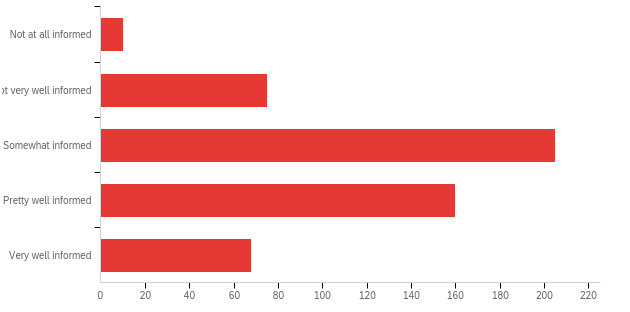


**Figure S2 ‘How informed do you feel about how sea level rise might impact on Aotearoa New Zealand?’ (n=519)**


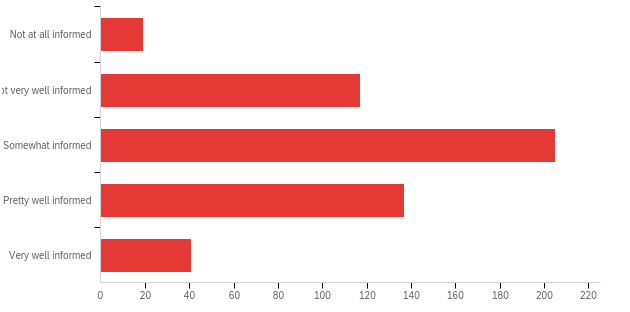


**Figure S3 ‘Given how informed you feel about sea level rise and how it might impact on Aotearoa New Zealand, how clear do you feel about the amount and rate and timing of sea level rise that will impact Aotearoa New Zealand to 2100?’ (n=518)**


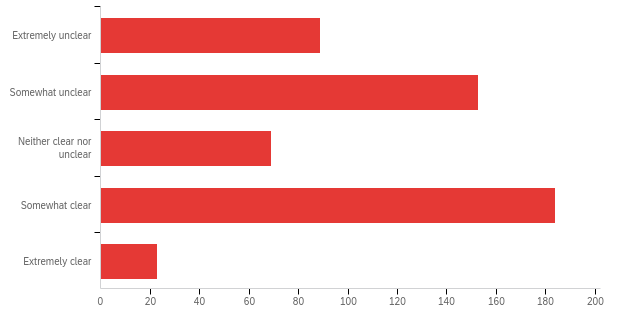


We then asked who they most trusted as a source of information about the amount, rate and timing of sea-level rise that will impact Aotearoa New Zealand this century. The majority (63.85%; n=520) selected ‘scientists’ as the best source of information about sea-level rise followed by ‘the IPCC’ (18.65%), ‘NGOs’ (4.62%), ‘central government’ (3.08%), ‘local government’ (1.35%) and last on the list ‘the media’ which only 0.96% of respondents selected. When asked if they knew of any scientists or science organisations working on sea-level rise in New Zealand, most respondents said ‘no’ (54.81%, n=520). For those who said yes, and provided a name, several individuals or organisations involved in sea-level rise were named, such as NIWA, the Deep South Challenge, Tim Naish, and James Renwick. But other individuals not involved in research into sea-level rise, such as freshwater scientist Mike Joy, also got significant responses.

**2.2 Comparison with questions reported in the main survey**

Results for the six questions reported in this paper followed the same pattern in the pilot survey as in the main survey.

The erroneous association of melting sea ice with sea-level rise identified in the main survey was apparent here, though to a lesser extent. In the pilot survey, respondents were asked to select ‘all that apply’ rather than rank the major causes of sea-level rise. Listed here in order of how many responses each mechanism got are (1) melting ice sheets, (2) melting of land-based glaciers, (3) thermal expansion of the ocean, (4) melting sea ice, (5) increased slash and sediment runoff due to deforestation, (6), land subsidence in some areas. Other options got 42 or less responses and are not listed here.

They were then asked to rank the *three things* likely to contribute most to sea-level rise by 2100: the mechanisms with the most responses were (1) melting ice sheets, (2) melting sea ice, (3) thermal expansions of the oceans, and (4) melting of land based glaciers.

**2.3 Other questions**

The survey also included other questions.

Respondents were asked how concerned they felt about the sea-level rise projected to happen in Aotearoa New Zealand by 2100. Responses are shown in the figure below.

**Figure S4 ‘How concerned do you feel about the sea level rise projected to happen in Aotearoa New Zealand to 2100?’ (n=490)**


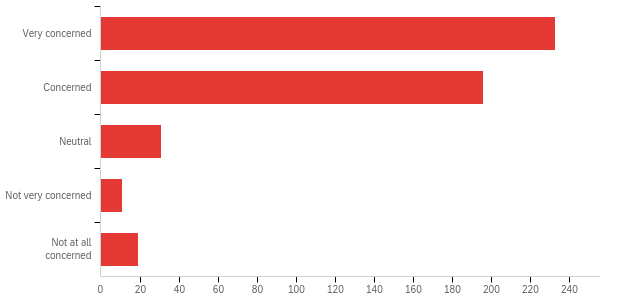


When asked ‘has your property been affected by sea-level rise?’, 94.9% of people said ‘no’. Only 3.88% of people said yes and the rest were unsure. When asked ‘has your business been affected by sea-level rise?’, 92.2% of people said ‘no’. Only 5.54% of people said yes and the rest were unsure.

When asked ‘have you been affected by sea-level rise in some other way?’, there were more positive responses. This time 21.79% of people said ‘yes’. When asked to ‘tell us about it’, respondents mentioned things such as coastal flooding, anxiety about sea-level rise, and rising insurance premiums. Most people (72.1%) answered ‘no’ to this question.

To gauge people’s understanding of the relationship between sea-level rise and coastal flooding, we asked: ‘After 50cm of sea level rise, how often do you think will we experience what is now a 1 in 100 year coastal flood?’ Responses are shown in the figure below.

**Figure S5: ‘After 50cm of sea level rise, how often do you think will we experience what is now a 1 in 100 year coastal flood?’ (n=487)**


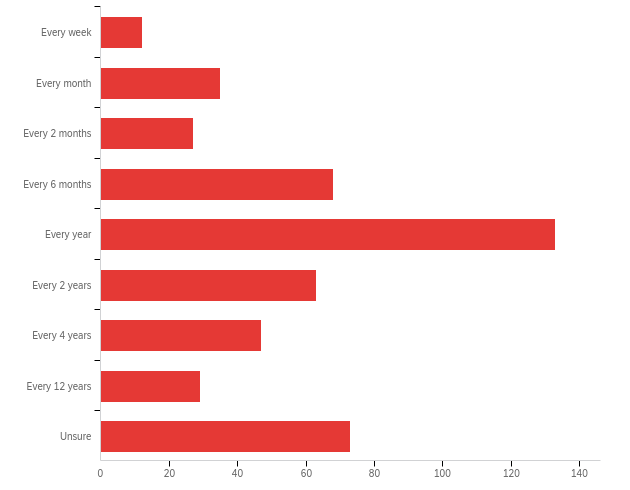


People were asked about the likelihood of a range of coastal impacts as a result of rising sea levels. Responses are shown in the table below.

**Table S4: ‘How likely (do you think) are the below impacts on coastal New Zealand as a result of rising sea levels?’**


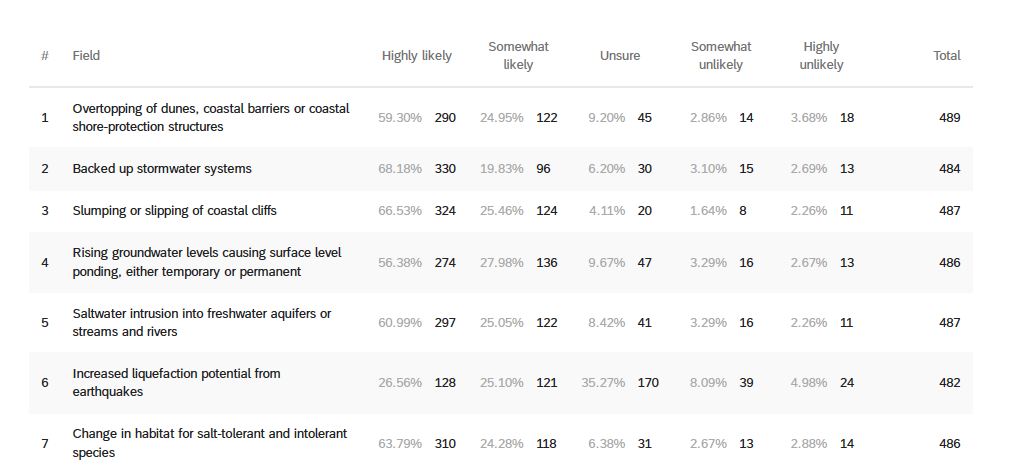


People were asked the extent to which they thought sea level rise was a result of climate change caused by human activities versus natural variation. Responses are shown in the figure below.

**Figure S6: ‘To what degree (do you think) is sea level rise a result of climate change caused by human activities versus natural variation?’ (n=488)**


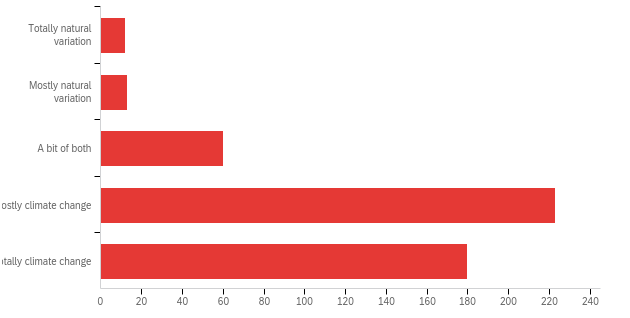


Finally, people were asked if they believed that sea level would continue to rise even if we cut carbon emissions to zero. Responses are shown in the figure below.

**Figure S7: ‘In your view, is the following statement true or false?: “If we cut carbon emissions today to zero, sea level will continue to rise for generations to come”’ (n=490)**


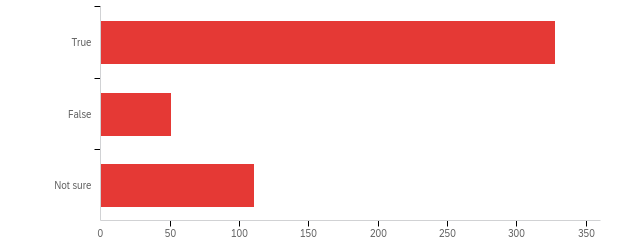


**3.0 References for Supporting information**

1. IPCC, 2019. Summary for Policymakers. In: Pörtner H-O, Roberts DC, Masson-Delmotte V, Zhai P, Tignor M, Poloczanska EK, et. al., editors. IPCC Special Report on the Ocean and Cryosphere in a Changing Climate. 2019. <https://www.ipcc.ch/srocc/chapter/summary-for-policymakers/>
2. Oppenheimer M, Glavovic BC, Hinkel J, van de Wal R, Magnan AK, Abd-Elgawad A, et al. Sea Level Rise and Implications for Low-Lying Islands, Coasts and Communities. In: Pörtner H-O, Roberts DC, Masson-Delmotte V, Zhai P, Tignor M, Poloczanska EK, et. al. editors. IPCC Special Report on the Ocean and Cryosphere in a Changing Climate.  2019. <https://www.ipcc.ch/srocc/chapter/chapter-4-sea-level-rise-and-implications-for-low-lying-islands-coasts-and-communities/>
3. Church JA, White NJ. Sea-Level Rise from the Late 19th to the Early 21st Century. Surveys in Geophysics. 2011; 32(4–5): 585–602. doi:10.1007/s10712-011-9119-1
4. Bamber J, Oppenheimer M, Kopp R, Aspinall W, Cooke R. Ice sheet contributions to future sea-level rise from structured expert judgment. PNAS. 2019; 23(116): 11195–11200. doi:10.1073/pnas.1817205116
5. Church J, Clark P, Cazenave A, Gregory J, Jevrejeva S, Levermann A, et. al. Sea level change. In: T. F. Stocker, D. Qin, G. K. Plattner, M. Tignor, S. K. Allen, J. Boschung, et. al., (editors), Climate change 2013: The physical science basis. Contribution of Working Group I to the Fifth Assessment Report of the Intergovernmental Panel on Climate Change. Cambridge: Cambridge University Press. 2013. <https://www.ipcc.ch/report/ar5/wg1/sea-level-change/>
6. Pfeffer WT, Harper J T, O’Neel S. Kinematic Constraints on Glacier Contributions to 21st-Century Sea-Level Rise. Science. 2008; 321(5894), 1340–1343. doi:10.1126/science.1159099
7. Kopp RE, Horton RM, Little CM, Mitrovica JX, Oppenheimer M, Rasmussen DJ, et. al. Probabilistic 21st and 22nd century sea-level projections at a global network of tide-gauge sites. Earth’s Future. 2014; 2(8): 383–406. doi:10.1002/2014EF000239
8. Hansen J, Sato M, Hearty P, Ruedy R, Kelley M, Masson-Delmotte V, et. al. Ice melt, sea level rise and superstorms: Evidence from paleoclimate data, climate modeling, and modern observations that 2 °C global warming could be dangerous. Atmospheric Chemistry and Physics. 2016; 16(6): 3761–3812. doi:10.5194/acp-16-3761-2016
9. Vaughan DG, Comiso JC, Allison I, Carrasco J, Kaser G, Kwok R, et. al. Observations: Cryosphere. In: Stocker TF, Qin D, Plattner G-K, Tignor M, Allen SK, Boschung J, editors. Climate Change 2013: The Physical Science Basis. Contribution of Working Group I to the Fifth Assessment Report of the Intergovernmental Panel on Climate Change. Cambridge: Cambridge University Press; 2013. <https://www.ipcc.ch/report/ar5/wg1/observations-cryosphere/>
10. Golledge NR, Kowalewski DE, Naish TR, Levy RH, Fogwill CJ, Gasson EGW. The multi-millennial Antarctic commitment to future sea-level rise. Nature. 2015; 526(7573): 421–425. doi:10.1038/nature15706
11. DeConto RM, Pollard D. Contribution of Antarctica to past and future sea-level rise. Nature. 2016; 531(7596): 591–597. doi:10.1038/nature17145
12. Hornsey MJ, Harris EA, Fielding, KS. Relationships among conspiratorial beliefs, conservatism and climate scepticism across nations. Nature Climate Change. 2018; 8(7): 614–620. doi:10.1038/s41558-018-0157-2

1. The response options in the study by Hornsey, Harris and Fielding (2018) were: ‘entirely caused by natural processes’, ‘mainly caused by natural processes’, ‘mainly caused by human activity’, ‘entirely caused by human activity’, ‘there is no such thing as climate change’ and ‘I don’t know’. The most common response in their sample was that climate change is ‘mainly’ or ‘entirely’ caused by human activity (comprising 72.4% of the sample). They created a continuous measure of climate change scepticism by coding ‘entirely caused by human activity’ as 1, ‘mainly caused by human activity’ as 2, ‘mainly caused by natural processes’ as 3, and ‘entirely caused by natural processes’ as 4. Only 1.5% of participants chose the option ‘There is no such thing as climate change’ and were also coded as 4, and respondents (4.9%) who clicked an option saying ‘I don’t know’ were excluded from their analyses. [↑](#footnote-ref-1)
